# Supplementary material for: Quantum-elevated chiral discrimination for biomolecules
Source: Sci Adv. 2026 Jan 14;12(3):eaea8201. doi: 10.1126/sciadv.aea8201 (PMC13267305; doi:10.1126/sciadv.aea8201)
Supplement: Supplementary file 1 — Supplementary Text S1 to S3 Figs. S1 to S10 References [file sciadv.aea8201_sm.pdf]

Science Advances
AAAS

Supplementary Materials for

Quantum-elevated chiral discrimination for biomolecules

Yiquan Yang *et al.*

Corresponding author: Guzhi Bao, guzhibao@sjtu.edu.cn; Weiping Zhang, wpz@sjtu.edu.cn

Sci. Adv. **12**, eaea8201 (2026)
DOI: 10.1126/sciadv.aea8201

This PDF file includes:

Supplementary Text S1 to S3
Figs. S1 to S10
References

Supplementary Text

1. Performance of two-mode squeezed state under loss

For the measurement parameter θ, we define the measurement precision $\delta\theta$ and the signal-to-noise ratio (SNR) ζ as:

$$\delta\theta = \sqrt{\frac{\delta^2 I_-}{\left(\partial_\theta I_-\right)^2}}, \quad \zeta = \frac{I_{\text{sig}}^2}{\delta^2 I_-}, \tag{S1}$$

where $I_- = \langle \hat{I}_- \rangle$, $I_{\text{sig}} = I_- - I_-|_{\theta=0}$ represents the signal, $\sqrt{\delta^2 I_-} = \sqrt{\langle \hat{I}_-^2 \rangle - \langle \hat{I}_- \rangle^2}$ characterizes the noise, and $\hat{I}_-$ is the physical observable. We further define the following quantum transformations within our theoretical model:

$$\begin{aligned}
&\hat{R}(\theta) = \begin{bmatrix} \cos\theta & \sin\theta \\ -\sin\theta & \cos\theta \end{bmatrix}, \\
&\hat{O}_{\text{BS}} = \frac{\sqrt{2}}{2}\begin{bmatrix} 1 & 1 \\ 1 & -1 \end{bmatrix}, \\
&\hat{O}_{\text{OPA}}\begin{bmatrix} \hat{M}_1 \\ \hat{M}_2 \end{bmatrix} = \begin{bmatrix} G\hat{M}_2 + g\hat{M}_1^\dagger \\ G\hat{M}_1 + g\hat{M}_2^\dagger \end{bmatrix}, \\
&\hat{O}_{\text{Loss}}\left\{\begin{bmatrix} \hat{M}_1 \\ \hat{M}_2 \end{bmatrix} \otimes \begin{bmatrix} \hat{V}_{\text{ac1}} \\ \hat{V}_{\text{ac2}} \end{bmatrix}\right\} = \begin{bmatrix} \sqrt{1-L}\hat{M}_1 + \sqrt{L}\hat{V}_{\text{ac1}} \\ \sqrt{1-L}\hat{M}_2 + \sqrt{L}\hat{V}_{\text{ac2}} \end{bmatrix},
\end{aligned} \tag{S2}$$

where $\hat{R}$ characterizes the polarization rotation, $\hat{O}_{\text{BS}}$ represents quantum operation of the 50:50 beam splitter (BS). $\hat{O}_{\text{OPA}}$ describes the optical parametric amplifier (OPA) process for the two input modes $\hat{M}_1$ and $\hat{M}_2$, with gain satisfying $G^2 - g^2 = 1$. $\hat{O}_{\text{Loss}}$ characterizes the loss of the modes $\hat{M}_1$ and $\hat{M}_2$, which are mixed with the vacuum mode $\hat{V}_{\text{ac1}}$ and $\hat{V}_{\text{ac2}}$, respectively.

Two-mode squeezed state (TMSS) can be generated by a non-degenerate OPA process. As demonstrated in Fig. S1, we consider the stimulated case, injected by vacuum state in the mode $\hat{a}_0$ and coherent state in the mode $\hat{b}_0$. Assuming the output modes $\hat{a}_1^{(L)}$ and $\hat{b}_1^{(L)}$ undergo the same propagation loss L before entering the detector. Based on Eq. (S2), we investigate the quantum noise performance of the photon number difference operator $\hat{I}_- = \hat{a}_1^{(L)\dagger}\hat{a}_1^{(L)} - \hat{b}_1^{(L)\dagger}\hat{b}_1^{(L)}$. The complete evolution process of the input modes $\hat{a}_0$ and $\hat{b}_0$ in Fig. S1 can be characterized by

$$\begin{bmatrix} \hat{a}_1^{(L)} \\ \hat{b}_1^{(L)} \end{bmatrix}_{\text{TMSS}} = \hat{O}_{\text{Loss}}\left\{\hat{O}_{\text{OPA}}\begin{bmatrix} \hat{b}_0 \\ \hat{a}_0 \end{bmatrix} \otimes \begin{bmatrix} \hat{d} \\ \hat{c} \end{bmatrix}\right\} = \begin{bmatrix} \sqrt{1-L}\left(G\hat{a}_0 + g\hat{b}_0^\dagger\right) + \sqrt{L}\hat{c} \\ \sqrt{1-L}\left(G\hat{b}_0 + g\hat{a}_0^\dagger\right) + \sqrt{L}\hat{d} \end{bmatrix}. \tag{S3}$$

To characterize the quantum enhancement, we compare it with coherent state (CS), which is shown in Fig. S2. The relationship between the input and output modes is described by

$$\begin{bmatrix} \hat{a}_1^{(L)} \\ \hat{b}_1^{(L)} \end{bmatrix}_{\text{CS}} = \begin{bmatrix} \sqrt{\frac{1-L}{2}}\left(\hat{a}_0 + \hat{b}_0\right) + \sqrt{L}\hat{c} \\ \sqrt{\frac{1-L}{2}}\left(\hat{b}_0 - \hat{a}_0\right) + \sqrt{L}\hat{d} \end{bmatrix}. \tag{S4}$$

Consider above two scenarios have identical total photon number

$$\left\langle \hat{I}_{\text{tot}} \right\rangle = \left\langle \hat{a}_1^{(L)\dagger}\hat{a}_1^{(L)} + \hat{b}_1^{(L)\dagger}\hat{b}_1^{(L)} \right\rangle = (1-L)\left[\left(2g^2+1\right)|\alpha|^2 + 2g^2\right] \tag{S5}$$

For the observable $\hat{I}_-$, their respective variances that characterize the quantum fluctuation are

$$\begin{aligned} \left(\delta^2 I_-\right)_{\text{TMSS}} &= (1-L)\left[\left(2g^2L+1\right)|\alpha|^2 + 2g^2L\right], \\ \left(\delta^2 I_-\right)_{\text{CS}} &= (1-L)\left[\left(2g^2+1\right)|\alpha|^2 + 2g^2\right]. \end{aligned} \tag{S6}$$

The numerical demonstration is depicted in Fig. S3. The quantum enhancement factor, defined as $(\delta^2 I_-)_{\text{CS}} / (\delta^2 I_-)_{\text{TMSS}}$, is shown in Fig. S4. In the absence of loss $(L=0)$, the enhancement factor reaches the maximum G^2+g^2 as $|\alpha| \gg g$. Quantum enhancement exists for any lossy parameter.

2. Performance of continuous-variable entangled state in chiral sensing under lossy conditions

We investigate the quantum enhancement enabled by continuous-variable polarization-entangled state (CVES) for chiral discrimination under lossy conditions, as illustrated in Fig. S5. Two OPAs are seeded with coherent states, $\left|\alpha/\sqrt{2}\right\rangle_H$ and $\left|\alpha/\sqrt{2}\right\rangle_V$, into their respective input modes $\hat{b}_{0,H}$ and $\hat{b}_{0,V}$. The signal (red) and idler (blue) beams are coherently mixed at PBS4 and PBS2. The generated CVES serves as a chiral probe and is directed through a chiral solution cell, which induces the polarization rotation to the probe. Then, the idler and signal modes are sent to PBS3 and PBS5 for detection. Two half-wave plates (HWP1 and HWP2), with the optical axis aligned along the horizontal direction, are placed before chiral solution cell. By rotating the HWPs clockwise or counterclockwise, the chiral signal can be nullified, allowing discrimination between L- and D-enantiomers. The evolution process of the input modes is characterized by

$$
\begin{aligned}
&\begin{bmatrix}\hat{a}_{1,H}\\ \hat{b}_{1,H}\end{bmatrix}=\hat{O}_{\text{OPA}}\begin{bmatrix}\hat{b}_{0,H}\\ \hat{a}_{0,H}\end{bmatrix},\begin{bmatrix}\hat{a}_{1,V}\\ \hat{b}_{1,V}\end{bmatrix}=\hat{O}_{\text{OPA}}\begin{bmatrix}\hat{b}_{0,V}\\ \hat{a}_{0,V}\end{bmatrix},\\
&\begin{bmatrix}\hat{a}_{2,H}^{(L)}\\ \hat{b}_{2,H}^{(L)}\end{bmatrix}=\hat{O}_{\text{Loss}}\left\{\begin{bmatrix}\hat{a}_{1,H}\\ \hat{b}_{1,H}\end{bmatrix}\otimes\begin{bmatrix}\hat{c}_{H}\\ \hat{d}_{H}\end{bmatrix}\right\},\begin{bmatrix}\hat{a}_{2,V}^{(L)}\\ \hat{b}_{2,V}^{(L)}\end{bmatrix}=\hat{O}_{\text{Loss}}\left\{\begin{bmatrix}-\hat{a}_{1,V}\\ \hat{b}_{1,V}\end{bmatrix}\otimes\begin{bmatrix}\hat{c}_{V}\\ \hat{d}_{V}\end{bmatrix}\right\},\\
&\begin{bmatrix}\hat{a}_{3,H}^{(L)}\\ \hat{a}_{3,V}^{(L)}\end{bmatrix}_{\text{CVES}}=\hat{R}(\theta)\begin{bmatrix}\hat{a}_{2,H}^{(L)}\\ \hat{a}_{2,V}^{(L)}\end{bmatrix}\\
&=\begin{bmatrix}\cos\theta\left[\sqrt{1-L}\left(G\hat{a}_{0,H}+g\hat{b}_{0,H}^{\dagger}\right)+\sqrt{L}\hat{c}_{H}\right]-\sin\theta\left[\sqrt{1-L}\left(G\hat{a}_{0,V}+g\hat{b}_{0,V}^{\dagger}\right)-\sqrt{L}\hat{c}_{V}\right]\\ -\cos\theta\left[\sqrt{1-L}\left(G\hat{a}_{0,V}+g\hat{b}_{0,V}^{\dagger}\right)-\sqrt{L}\hat{c}_{V}\right]-\sin\theta\left[\sqrt{1-L}\left(G\hat{a}_{0,H}+g\hat{b}_{0,H}^{\dagger}\right)+\sqrt{L}\hat{c}_{H}\right]\end{bmatrix},\\
&\begin{bmatrix}\hat{b}_{3,H}^{(L)}\\ \hat{b}_{3,V}^{(L)}\end{bmatrix}_{\text{CVES}}=\hat{R}(\theta)\begin{bmatrix}\hat{b}_{2,H}^{(L)}\\ \hat{b}_{2,V}^{(L)}\end{bmatrix}\\
&=\begin{bmatrix}\cos\theta\left[\sqrt{1-L}\left(G\hat{b}_{0,H}+g\hat{a}_{0,H}^{\dagger}\right)+\sqrt{L}\hat{d}_{H}\right]+\sin\theta\left[\sqrt{1-L}\left(G\hat{b}_{0,V}+g\hat{a}_{0,V}^{\dagger}\right)+\sqrt{L}\hat{d}_{V}\right]\\ \cos\theta\left[\sqrt{1-L}\left(G\hat{b}_{0,V}+g\hat{a}_{0,V}^{\dagger}\right)+\sqrt{L}\hat{d}_{V}\right]-\sin\theta\left[\sqrt{1-L}\left(G\hat{b}_{0,H}+g\hat{a}_{0,H}^{\dagger}\right)+\sqrt{L}\hat{d}_{H}\right]\end{bmatrix}.
\end{aligned}
\tag{S7}
$$

For comparison, chiral discrimination with a coherent state is depicted in Fig. S6, in which the operator evolution follows

$$
\begin{aligned}
&\begin{bmatrix}\hat{a}_{3,H}^{(L)}\\ \hat{a}_{3,V}^{(L)}\end{bmatrix}_{\text{CS}}=\begin{bmatrix}\cos\theta\left[\sqrt{1-L}\,\dfrac{\hat{a}_{0,H}+\hat{b}_{0,H}}{\sqrt{2}}+\sqrt{L}\hat{c}_{H}\right]-\sin\theta\left[\sqrt{1-L}\,\dfrac{\hat{a}_{0,V}+\hat{b}_{0,V}}{\sqrt{2}}-\sqrt{L}\hat{c}_{V}\right]\\ -\cos\theta\left[\sqrt{1-L}\,\dfrac{\hat{a}_{0,V}+\hat{b}_{0,V}}{\sqrt{2}}-\sqrt{L}\hat{c}_{V}\right]-\sin\theta\left[\sqrt{1-L}\,\dfrac{\hat{a}_{0,H}+\hat{b}_{0,H}}{\sqrt{2}}+\sqrt{L}\hat{c}_{H}\right]\end{bmatrix},\\
&\begin{bmatrix}\hat{b}_{3,H}^{(L)}\\ \hat{b}_{3,V}^{(L)}\end{bmatrix}_{\text{CS}}=\begin{bmatrix}\cos\theta\left[\sqrt{1-L}\,\dfrac{\hat{b}_{0,H}-\hat{a}_{0,H}}{\sqrt{2}}+\sqrt{L}\hat{d}_{H}\right]+\sin\theta\left[\sqrt{1-L}\,\dfrac{\hat{b}_{0,V}-\hat{a}_{0,V}}{\sqrt{2}}+\sqrt{L}\hat{d}_{V}\right]\\ \cos\theta\left[\sqrt{1-L}\,\dfrac{\hat{b}_{0,V}-\hat{a}_{0,V}}{\sqrt{2}}+\sqrt{L}\hat{d}_{V}\right]-\sin\theta\left[\sqrt{1-L}\,\dfrac{\hat{b}_{0,H}-\hat{a}_{0,H}}{\sqrt{2}}+\sqrt{L}\hat{d}_{H}\right]\end{bmatrix}.
\end{aligned}
\tag{S8}
$$

We take the operator $\hat{I}_{-}=(\hat{a}_{3,V}^{(L)\dagger}\hat{a}_{3,V}^{(L)}-\hat{a}_{3,H}^{(L)\dagger}\hat{a}_{3,H}^{(L)})-(\hat{b}_{3,V}^{(L)\dagger}\hat{b}_{3,V}^{(L)}-\hat{b}_{3,H}^{(L)\dagger}\hat{b}_{3,H}^{(L)})$ as the observable for chiral discrimination. Under the condition of identical total photon number, i.e. $\langle\hat{I}_{\text{tot}}\rangle=\langle\hat{a}_{3,H}^{(L)\dagger}\hat{a}_{3,H}^{(L)}+\hat{a}_{3,V}^{(L)\dagger}\hat{a}_{3,V}^{(L)}+\hat{b}_{3,H}^{(L)\dagger}\hat{b}_{3,H}^{(L)}+\hat{b}_{3,V}^{(L)\dagger}\hat{b}_{3,V}^{(L)}\rangle=(1-L)[(2g^2+1)|\alpha|^2+4g^2]$, we derive the signal and noise for CVES and CS under the condition $|\theta|\ll 1$, $g\gg 1$ and $|\alpha|\gg 1$, which are

$$
\begin{aligned}
&\left(I_{\text{sig}}\right)_{\text{CVES}} = 2(1-L)\left(2g^2+1\right)|\alpha|^2\,\theta = 2\langle \hat{I}_{\text{tot}}\rangle\theta,\\
&\left(I_{\text{sig}}\right)_{\text{CS}} = 2(1-L)\left[\left(2g^2+1\right)|\alpha|^2+4g^2\right]\theta = 2\langle \hat{I}_{\text{tot}}\rangle\theta,\\
&\left(\sqrt{\delta^2 I_-}\right)_{\text{CVES}} = \sqrt{(1-L)\left[\left(2g^2L+1\right)|\alpha|^2+4g^2L\right]} = \sqrt{\langle \hat{I}_{\text{tot}}\rangle\left(\frac{1}{G^2+g^2}+L\right)},\\
&\left(\sqrt{\delta^2 I_-}\right)_{\text{CS}} = \sqrt{(1-L)\left[\left(2g^2+1\right)|\alpha|^2+4g^2\right]} = \sqrt{\langle \hat{I}_{\text{tot}}\rangle}.
\end{aligned}
\tag{S9}
$$

And we obtain the SNR and sensitivity for CVES and CS as follows

$$
\begin{aligned}
&\zeta_{\text{CVES}} = \frac{4(1-L)\left(2g^2+1\right)^2|\alpha|^4}{\left(2g^2L+1\right)|\alpha|^2+4g^2L}\theta^2 = 4\langle \hat{I}_{\text{tot}}\rangle\left(\frac{1}{G^2+g^2}+L\right)^{-1}\theta^2,\\
&\zeta_{\text{CS}} = 4(1-L)\left[\left(2g^2+1\right)|\alpha|^2+4g^2\right]\theta^2 = 4\langle \hat{I}_{\text{tot}}\rangle\theta^2,\\
&\delta\theta_{\text{CVES}} = \sqrt{\frac{\left(2g^2L+1\right)|\alpha|^2+4g^2L}{4(1-L)\left(2g^2+1\right)^2|\alpha|^4}} = \frac{1}{2}\sqrt{\frac{1}{\langle \hat{I}_{\text{tot}}\rangle}\left(\frac{1}{G^2+g^2}+L\right)},\\
&\delta\theta_{\text{CS}} = \sqrt{\frac{1}{4(1-L)\left[\left(2g^2+1\right)|\alpha|^2+4g^2\right]}} = \frac{1}{2}\frac{1}{\sqrt{\langle \hat{I}_{\text{tot}}\rangle}}.
\end{aligned}
\tag{S10}
$$

The numerical demonstrations are shown in Fig. S7 and Fig. S8, revealing that the entangled scheme outperforms its classical counterpart for arbitrary loss. In the losses limit for $|\alpha| \gg g$, the enhancement factor, defined as $\zeta_{\text{CVES}}/\zeta_{\text{CS}} = (\delta\theta_{\text{CS}}/\delta\theta_{\text{CVES}})^2$, approaches G^2+g^2.

To surpass the shot-noise limit (SNL), the total propagation loss must be below a critical threshold, which exists for both CV and discrete-variable (DV) entangled states. We compare the loss thresholds for the CVES and DV N00N state. The thresholds are given by (*82, 83*)

$$
\begin{aligned}
&L_{\text{CVES}} = \frac{2g^2|\alpha|^2\left[\left(2g^2+1\right)|\alpha|^2-2\right]}{\left(8g^4+6g^2+1\right)|\alpha|^4+4\left(4g^4+g^2\right)|\alpha|^2+16g^4},\\
&L_{\text{N00N}} = 1-\sqrt[N]{1/N},
\end{aligned}
\tag{S11}
$$

where N is the photon number of N00N state, the total photon number for the CVES is $(2g^2+1)|\alpha|^2+4g^2$. As shown in the numerical results in Fig. S9, the CVES exhibits significantly greater robustness and higher tolerance to loss compared to the DV N00N state.

3. Phase stabilization

To generate high-quality CVES for chiral discrimination, phase stabilization between the idler modes $\hat{a}_{2,H}^{(L)}$ and $\hat{a}_{2,V}^{(L)}$ (as well as signal modes $\hat{b}_{2,H}^{(L)}$ and $\hat{b}_{2,V}^{(L)}$), produced by OPA1 and OPA2, is essential. The schematic is shown in Fig. S10. Leakage light from the signal and idler beams passes through a HWP with its fast axis at an angle of $\pi/8$ relative to the horizontal direction and is then detected by a balanced amplified photodetector. In the Heisenberg picture, the annihilation operators $\hat{a}_H$ and $\hat{a}_V$ for horizontally and vertically polarized modes evolve as

$$\begin{bmatrix} \hat{a}'_H \\ \hat{a}'_V \end{bmatrix} = \hat{R}(\pi/8) \begin{bmatrix} 1 & 0 \\ 0 & i \end{bmatrix} \hat{R}(-\pi/8) \begin{bmatrix} 1 & 0 \\ 0 & e^{i\phi_j} \end{bmatrix} \begin{bmatrix} \hat{a}_H \\ \hat{a}_V \end{bmatrix} = \begin{bmatrix} (1+e^{i\phi_j})\hat{a}_H \\ (1-e^{i\phi_j})\hat{a}_V \end{bmatrix}, \tag{S12}$$

where ϕ_j is the relative phase between H and V modes, which must be actively stabilized. The differential intensity signal, $\left\langle \hat{a}'^{\dagger}_H \hat{a}'_H - \hat{a}'^{\dagger}_V \hat{a}'_V \right\rangle$, serves as error signal, which is proportional to $\cos\phi_j$. This signal is used in a feedback loop to lock the relative phase at $\phi_j = (-1)^{j+1}\pi/2$, where the sign is determined by the positivity of the PID gain. In the experiment, the signal and idler phases are deliberately stabilized at $\pi/2$ and $-\pi/2$, respectively, to enhance the phase-sensitive signal relevant to chiral discrimination.

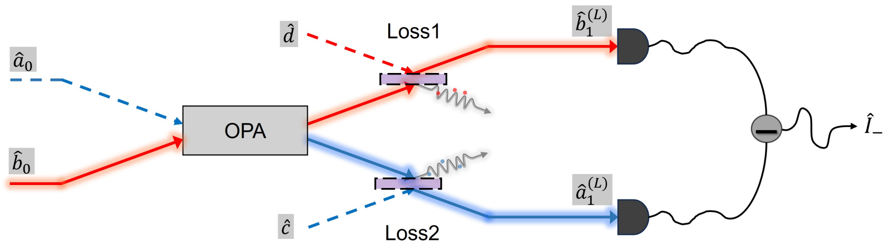

Fig. S1 Loss model of two-mode squeezed state (TMSS). The TMSS is generated by stimulated optical parametric amplifier (OPA).

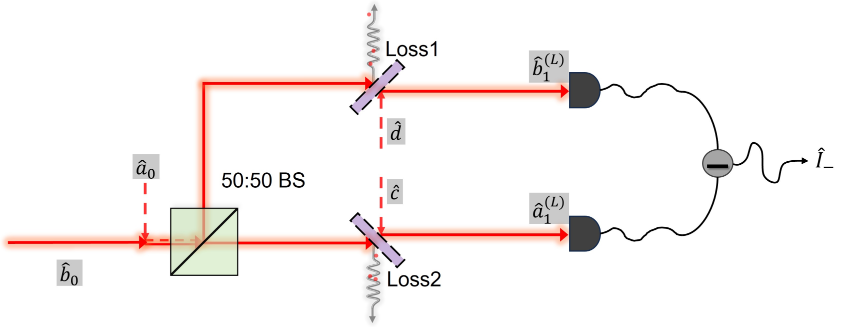

Fig. S2 Loss model of coherent state. This model is built by replacing optical parametric amplifier in Fig. S1 with balanced beam splitter (BS).

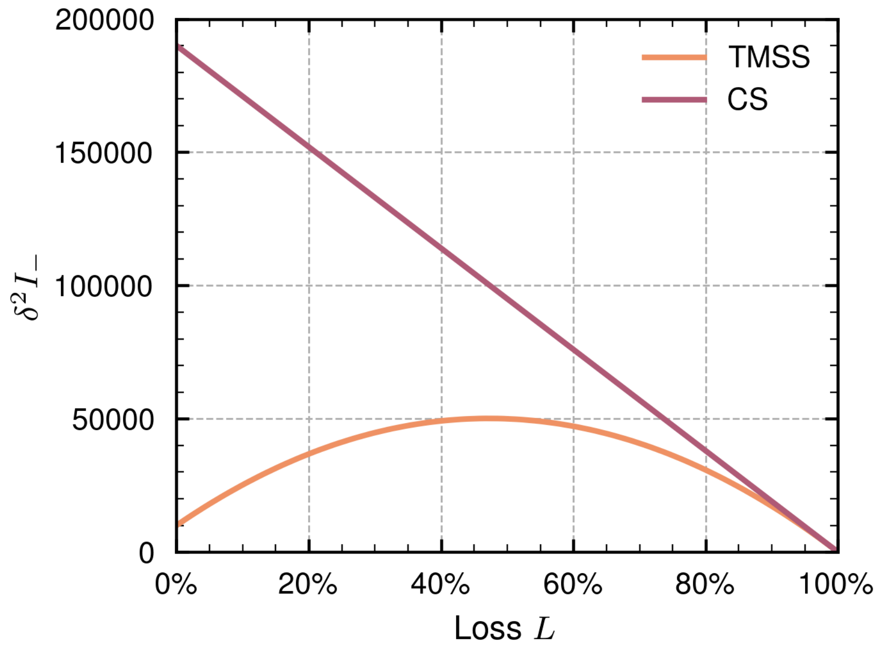

Fig. S3 Variance comparison between two-mode squeezed state (TMSS) and coherent state (CS) of the observable $\hat{I}_-$ under lossy conditions. We set $|\alpha|=100$ and $g=3$.

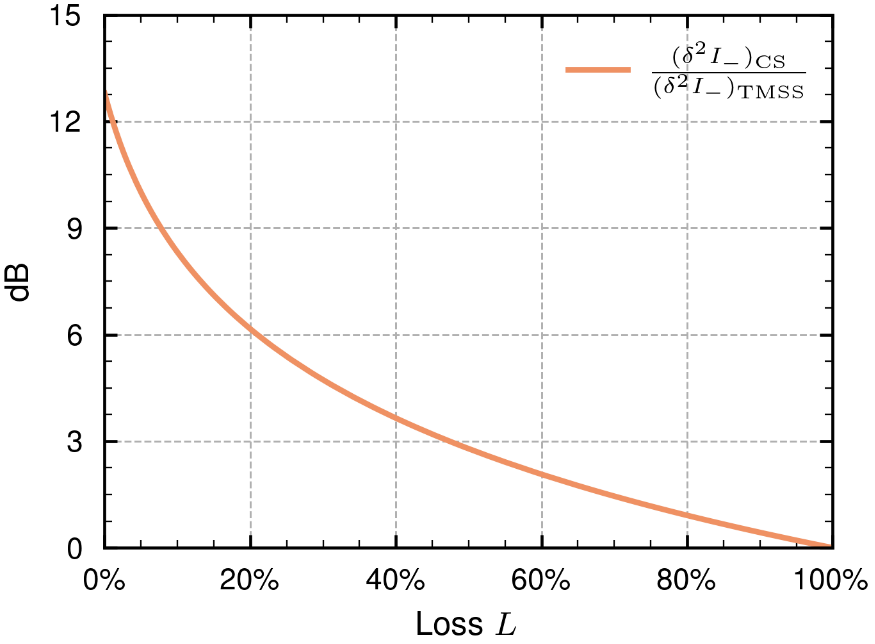

Fig. S4 Logarithmic quantum enhancement factor of the two-mode squeezed state (TMSS) under lossy conditions. The Logarithmic quantum enhancement factor is defined as $10\log[(\delta^2 I_-)_{CS} / (\delta^2 I_-)_{TMSS}]$. CS: coherent state.

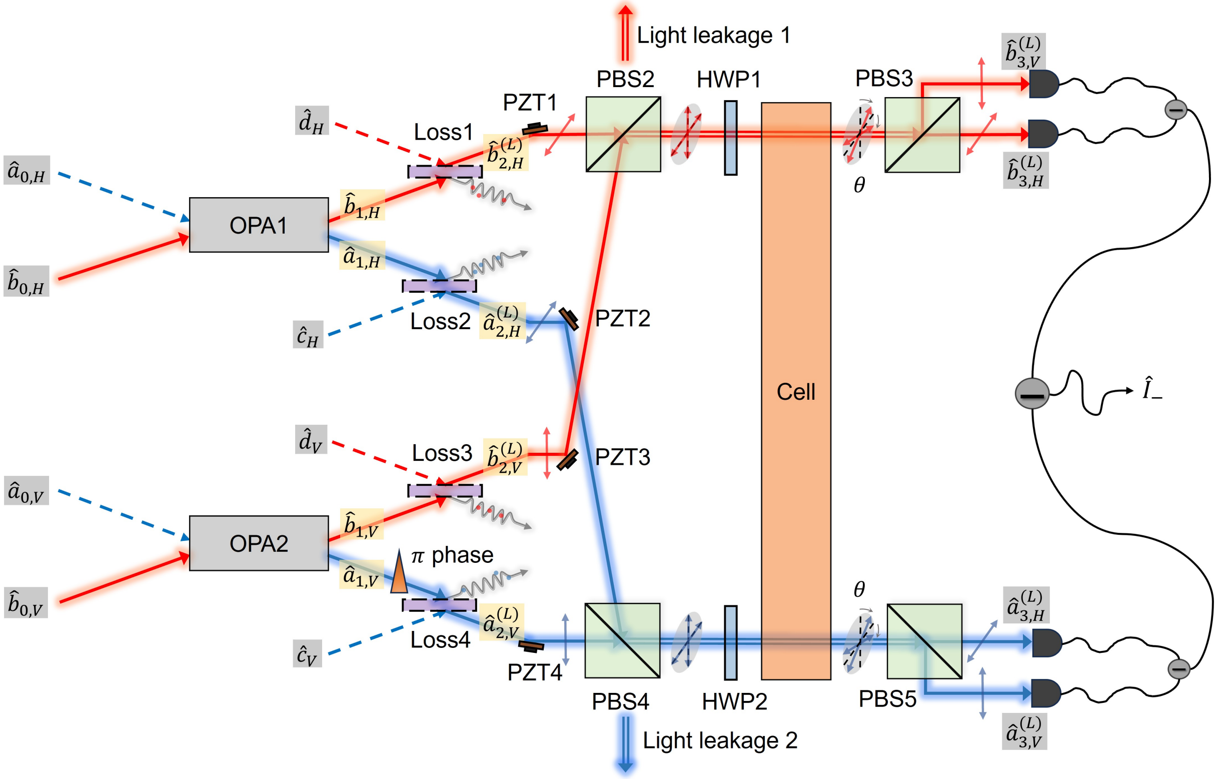

Fig. S5 Chiral discrimination with continuous-variable polarization-entangled state under lossy conditions. OPA: optical parametric amplifier. PBS: polarized beam splitter. HWP: half-wave plate. PZT: piezoelectric transducer. The leakage light at PBS2 and PBS4 is due to non-perfect polarization extinction ratio.

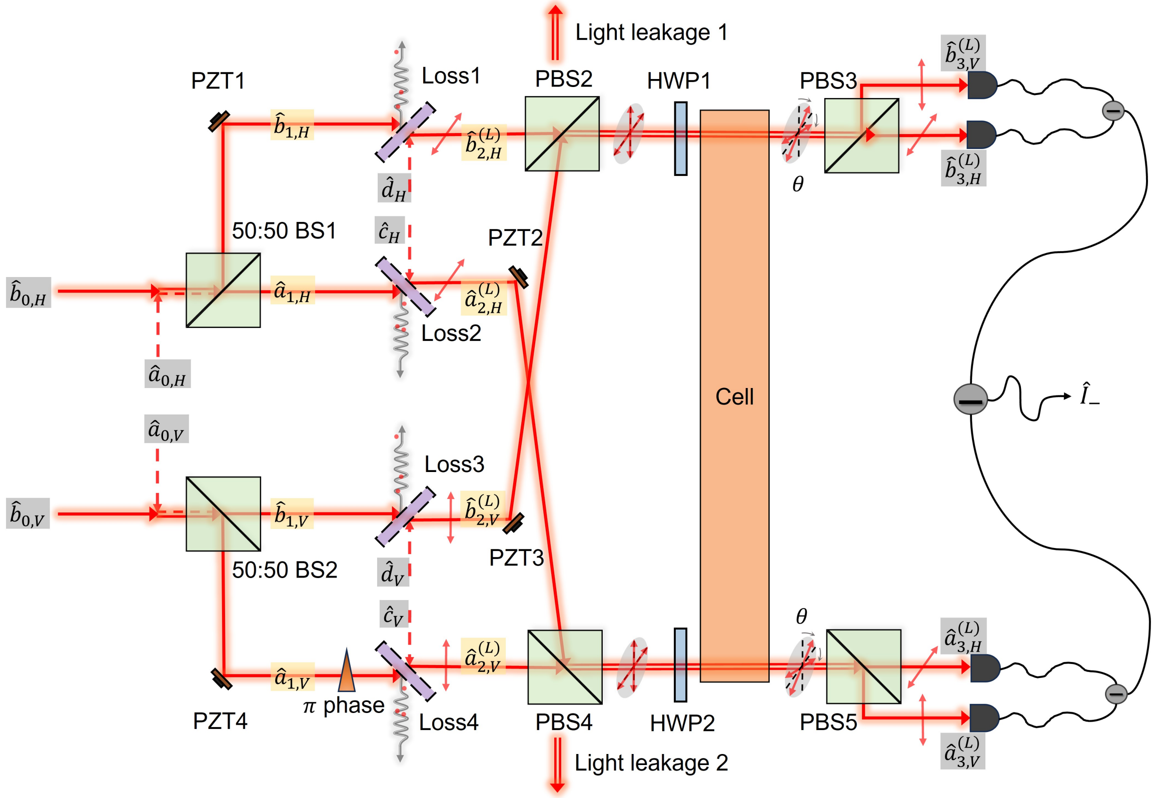

Fig. S6 Chiral discrimination with coherent state under lossy conditions. BS: beam splitter. PBS: polarized beam splitter. HWP: half-wave plate. PZT: piezoelectric transducer. The leakage light at PBS2 and PBS4 is due to non-perfect polarization extinction ratio.

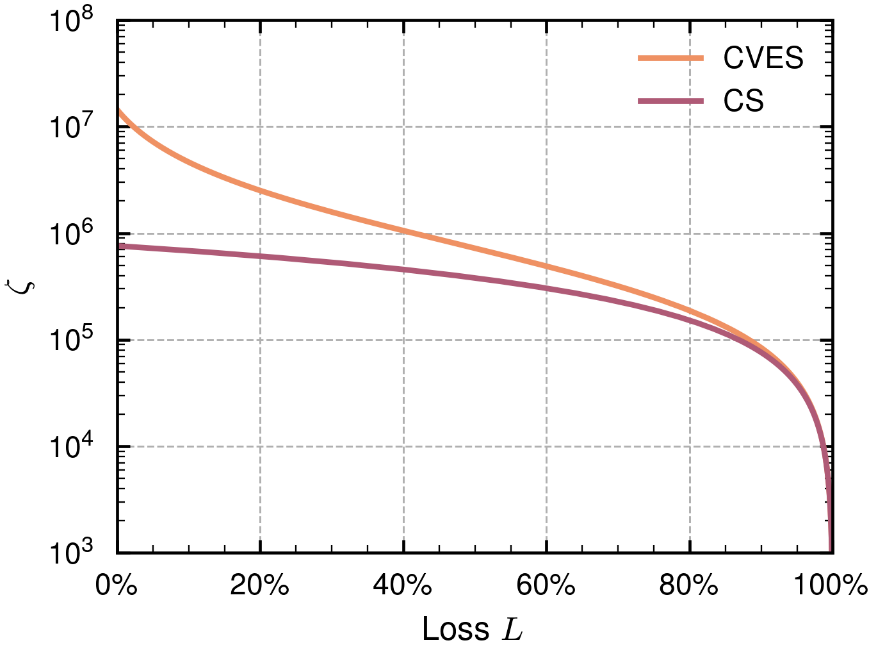

Fig. S7 SNR comparison between continuous-variable entangled state (CVES) and coherent state (CS) under lossy conditions. We set $|\alpha|=100$, $g=3$ and $\theta=1^{\circ}$.

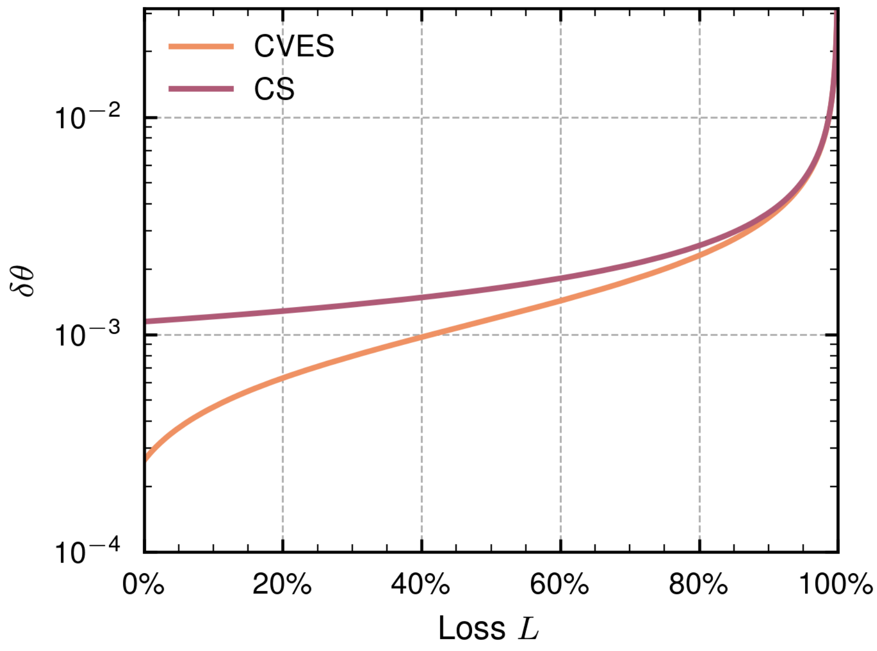

Fig. S8 Sensitivity comparison between continuous-variable entangled state (CVES) and coherent state (CS) under lossy conditions. We set $|\alpha|=100$ and $g=3$.

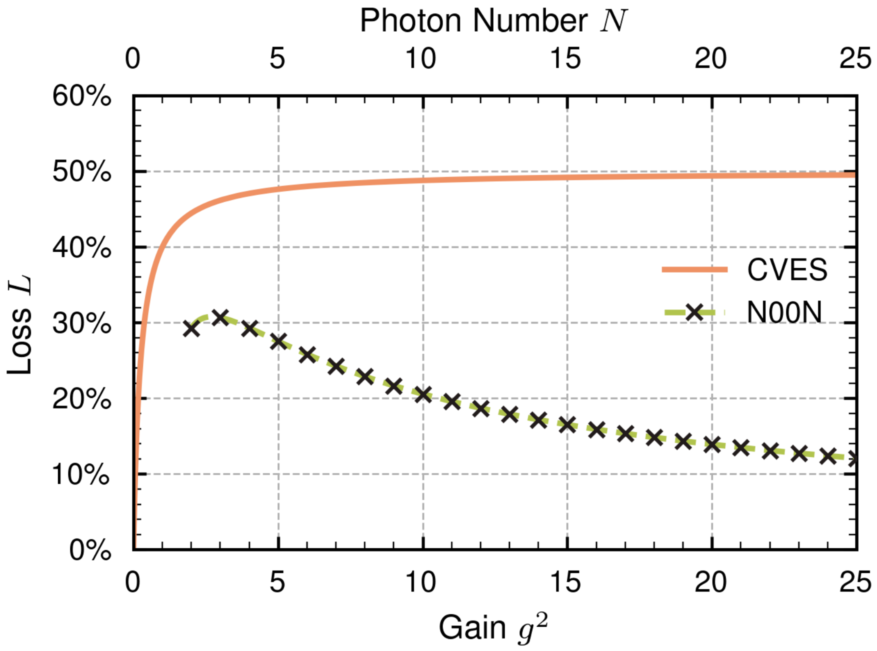

Fig. S9 Loss threshold comparison between continuous-variable entangled state (CVES) and N00N state. We set $|\alpha|=100$.

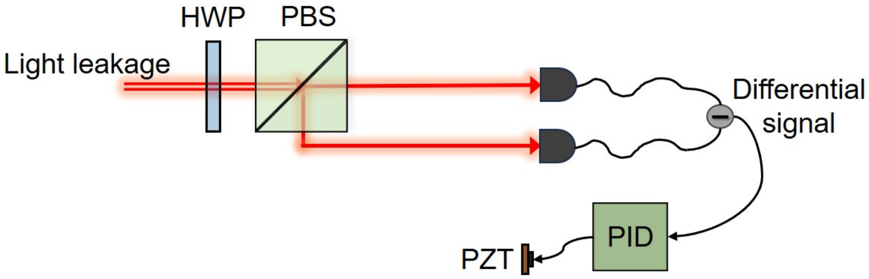

Fig. S10 Phase stabilization scheme. We set $|\alpha|=100$. The leakage light passes through a half-wave plate (HWP) and is subsequently split by a polarizing beam splitter (PBS) for differential intensity measurement. The resulting signal serves as an error signal, which is processed by a proportional-integral-derivative (PID) controller to generate a phase feedback signal applied to a piezoelectric transducer (PZT) mounted on the mirror.

REFERENCES

1. L. D. Barron, Symmetry and molecular chirality. *Chem. Soc. Rev.* **15**, 189 (1986).
2. L. A. Nguyen, H. He, C. Pham-Huy, Chiral drugs. An overview. *Int. J. Biomed. Sci.* **2**, 85–100 (2006).
3. G. Lin, Q. You, J. Cheng, Eds., *Chiral Drugs: Chemistry and Biological Action* (Wiley, ed. 1, 2011).
4. J. Kumar, H. Eraña, E. López-Martínez, N. Claes, V. F. Martín, D. M. Solís, S. Bals, A. L. Cortajarena, J. Castilla, L. M. Liz-Marzán, Detection of amyloid fibrils in Parkinson's disease using plasmonic chirality. *Proc. Natl. Acad. Sci. U.S.A.* **115**, 3225–3230 (2018).
5. Y. Liu, Z. Wu, D. W. Armstrong, H. Wolosker, Y. Zheng, Detection and analysis of chiral molecules as disease biomarkers. *Nat. Rev. Chem.* **7**, 355–373 (2023).
6. P. L. Polavarapu, Optical rotation: Recent advances in determining the absolute configuration. *Chirality* **14**, 768–781 (2002).
7. E. Castiglioni, S. Abbate, G. Longhi, Experimental methods for measuring optical rotatory dispersion: Survey and outlook. *Chirality* **23**, 711–716 (2011).
8. N. Berova, L. D. Bari, G. Pescitelli, Application of electronic circular dichroism in configurational and conformational analysis of organic compounds. *Chem. Soc. Rev.* **36**, 914 (2007).
9. P. J. Stephens, F. J. Devlin, J. Pan, The determination of the absolute configurations of chiral molecules using vibrational circular dichroism (VCD) spectroscopy. *Chirality* **20**, 643–663 (2008).
10. G. Pescitelli, L. Di Bari, N. Berova, Application of electronic circular dichroism in the study of supramolecular systems. *Chem. Soc. Rev.* **43**, 5211–5233 (2014).
11. P. L. Polavarapu, E. Santoro, Vibrational optical activity for structural characterization of natural products. *Nat. Prod. Rep.* **37**, 1661–1699 (2020).

12. L. D. Barron, L. Hecht, I. H. McColl, E. W. Blanch, Raman optical activity comes of age. *Mol. Phys.* **102**, 731–744 (2004).

13. M. Krupová, J. Kessler, P. Bouř, Recent trends in chiroptical spectroscopy: Theory and applications of vibrational circular dichroism and raman optical activity. *ChemPlusChem* **85**, 561–575 (2020).

14. A. M. Edwards, E. Silva, Effect of visible light on selected enzymes, vitamins and amino acids. *J. Photochem. Photobiol. B Biol.* **63**, 126–131 (2001).

15. C. K. Remucal, K. McNeill, Photosensitized amino acid degradation in the presence of riboflavin and its derivatives. *Environ. Sci. Technol.* **45**, 5230–5237 (2011).

16. G. Yan, L. Zhang, C. Feng, R. Gong, E. Idiiatullina, Q. Huang, M. He, S. Guo, F. Yang, Y. Li, F. Ding, W. Ma, V. Pavlov, Z. Han, Z. Wang, C. Xu, B. Cai, Y. Yuan, L. Yang, Blue light emitting diodes irradiation causes cell death in colorectal cancer by inducing ROS production and DNA damage. *Int. J. Biochem. Cell Biol.* **103**, 81–88 (2018).

17. Y. Tang, A. E. Cohen, Optical chirality and its interaction with matter. *Phys. Rev. Lett.* **104**, 163901 (2010).

18. E. Hendry, T. Carpy, J. Johnston, M. Popland, R. V. Mikhaylovskiy, A. J. Lapthorn, S. M. Kelly, L. D. Barron, N. Gadegaard, M. Kadodwala, Ultrasensitive detection and characterization of biomolecules using superchiral fields. *Nat. Naotech.* **5**, 783–787 (2010).

19. Y. Zhao, A. N. Askarpour, L. Sun, J. Shi, X. Li, A. Alù, Chirality detection of enantiomers using twisted optical metamaterials. *Nat. Commun.* **8**, 14180 (2017).

20. Y. Y. Lee, R. M. Kim, S. W. Im, M. Balamurugan, K. T. Nam, Plasmonic metamaterials for chiral sensing applications. *Nanoscale* **12**, 58–66 (2020).

21. X. Mu, L. Hu, Y. Cheng, Y. Fang, M. Sun, Chiral surface plasmon-enhanced chiral spectroscopy: Principles and applications. *Nanoscale* **13**, 581–601 (2021).

22. J. García-Guirado, M. Svedendahl, J. Puigdollers, R. Quidant, Enhanced chiral sensing with dielectric nanoresonators. *Nano Lett.* **20**, 585–591 (2020).

23. L. A. Warning, A. R. Miandashti, L. A. McCarthy, Q. Zhang, C. F. Landes, S. Link, Nanophotonic approaches for chirality sensing. *ACS Nano* **15**, 15538–15566 (2021).

24. M. Hentschel, M. Schäferling, X. Duan, H. Giessen, N. Liu, Chiral plasmonics. *Sci. Adv.* **3**, e1602735 (2017).

25. D. Sofikitis, L. Bougas, G. E. Katsoprinakis, A. K. Spiliotis, B. Loppinet, T. P. Rakitzis, Evanescent-wave and ambient chiral sensing by signal-reversing cavity ringdown polarimetry. *Nature* **514**, 76–79 (2014).

26. L. Bougas, J. Byron, D. Budker, J. Williams, Absolute optical chiral analysis using cavity-enhanced polarimetry. *Sci. Adv.* **8**, eabm3749 (2022).

27. W. Zhou, Y.-P. Ruan, H. Wu, H. Zhang, J.-S. Tang, Z. Xie, L. Tang, Y. Wang, Y.-E. Ji, K. Jia, C.-W. Qiu, Y.-Q. Lu, K. Xia, Magnetic-free chiral eigenmode spectroscopy for simultaneous sensitive measurement of optical rotary dispersion and circular dichroism. *eLight* **4**, 12 (2024).

28. E. Mohammadi, K. L. Tsakmakidis, A. N. Askarpour, P. Dehkhoda, A. Tavakoli, H. Altug, Nanophotonic platforms for enhanced chiral sensing. *ACS Photonics* **5**, 2669–2675 (2018).

29. S. Yoo, Q.-H. Park, Metamaterials and chiral sensing: A review of fundamentals and applications. *Nanophotonics* **8**, 249–261 (2019).

30. C. He, G. Yang, Y. Kuai, S. Shan, L. Yang, J. Hu, D. Zhang, Q. Zhang, G. Zou, Dissymmetry enhancement in enantioselective synthesis of helical polydiacetylene by application of superchiral light. *Nat. Commun.* **9**, 5117 (2018).

31. D. Ayuso, O. Neufeld, A. F. Ordonez, P. Decleva, G. Lerner, O. Cohen, M. Ivanov, O. Smirnova, Synthetic chiral light for efficient control of chiral light–matter interaction. *Nat. Photonics* **13**, 866–871 (2019).

32. W. Brullot, M. K. Vanbel, T. Swusten, T. Verbiest, Resolving enantiomers using the optical angular momentum of twisted light. *Sci. Adv.* **2**, e1501349 (2016).

33. J. Ni, S. Liu, D. Wu, Z. Lao, Z. Wang, K. Huang, S. Ji, J. Li, Z. Huang, Q. Xiong, Y. Hu, J. Chu, C.-W. Qiu, Gigantic vortical differential scattering as a monochromatic probe for multiscale chiral structures. *Proc. Natl. Acad. Sci. U.S.A.* **118**, e2020055118 (2021).

34. J.-L. Bégin, A. Jain, A. Parks, F. Hufnagel, P. Corkum, E. Karimi, T. Brabec, R. Bhardwaj, Nonlinear helical dichroism in chiral and achiral molecules. *Nat. Photonics* **17**, 82–88 (2023).

35. N. Mayer, D. Ayuso, P. Decleva, M. Khokhlova, E. Pisanty, M. Ivanov, O. Smirnova, Chiral topological light for detection of robust enantiosensitive observables. *Nat. Photonics* **18**, 1155–1160 (2024).

36. R. Cireasa, A. E. Boguslavskiy, B. Pons, M. C. H. Wong, D. Descamps, S. Petit, H. Ruf, N. Thiré, A. Ferré, J. Suarez, J. Higuet, B. E. Schmidt, A. F. Alharbi, F. Légaré, V. Blanchet, B. Fabre, S. Patchkovskii, O. Smirnova, Y. Mairesse, V. R. Bhardwaj, Probing molecular chirality on a sub-femtosecond timescale. *Nat. Phys.* **11**, 654–658 (2015).

37. D. Baykusheva, D. Zindel, V. Svoboda, E. Bommeli, M. Ochsner, A. Tehlar, H. J. Wörner, Real-time probing of chirality during a chemical reaction. *Proc. Natl. Acad. Sci. U.S.A.* **116**, 23923–23929 (2019).

38. D. Ayuso, A. F. Ordonez, M. Ivanov, O. Smirnova, Ultrafast optical rotation in chiral molecules with ultrashort and tightly focused beams. *Optica* **8**, 1243 (2021).

39. D. Habibović, K. R. Hamilton, O. Neufeld, L. Rego, Emerging tailored light sources for studying chirality and symmetry. *Nat. Rev. Phys.* **6**, 663–675 (2024).

40. M. W. Mitchell, J. S. Lundeen, A. M. Steinberg, Super-resolving phase measurements with a multiphoton entangled state. *Nature* **429**, 161–164 (2004).

41. T. Nagata, R. Okamoto, J. L. O'Brien, K. Sasaki, S. Takeuchi, Beating the standard quantum limit with four-entangled photons. *Science* **316**, 726–729 (2007).

42. S. Slussarenko, M. M. Weston, H. M. Chrzanowski, L. K. Shalm, V. B. Verma, S. W. Nam, G. J. Pryde, Unconditional violation of the shot-noise limit in photonic quantum metrology. *Nat. Photonics* **11**, 700–703 (2017).

43. H. Defienne, B. Ndagano, A. Lyons, D. Faccio, Polarization entanglement-enabled quantum holography. *Nat. Phys.* **17**, 591–597 (2021).

44. R. Silvestri, H. Yu, T. Strömberg, C. Hilweg, R. W. Peterson, P. Walther, Experimental observation of Earth's rotation with quantum entanglement. *Sci. Adv.* **10**, eado0215 (2024).

45. N. Tischler, M. Krenn, R. Fickler, X. Vidal, A. Zeilinger, G. Molina-Terriza, Quantum optical rotatory dispersion. *Sci. Adv.* **2**, e1601306 (2016).

46. D. Ganapathy, W. Jia, M. Nakano, V. Xu, N. Aritomi, T. Cullen, N. Kijbunchoo, S. E. Dwyer, A. Mullavey, L. McCuller, R. Abbott, I. Abouelfettouh, R. X. Adhikari, A. Ananyeva, S. Appert, K. Arai, S. M. Aston, M. Ball, S. W. Ballmer, D. Barker, L. Barsotti, B. K. Berger, J. Betzwieser, D. Bhattacharjee, G. Billingsley, S. Biscans, N. Bode, E. Bonilla, V. Bossilkov, A. Branch, A. F. Brooks, D. D. Brown, J. Bryant, C. Cahillane, H. Cao, E. Capote, F. Clara, J. Collins, C. M. Compton, R. Cottingham, D. C. Coyne, R. Crouch, J. Csizmazia, L. P. Dartez, N. Demos, E. Dohmen, J. C. Driggers, A. Effler, A. Ejlli, T. Etzel, M. Evans, J. Feicht, R. Frey, W. Frischhertz, P. Fritschel, V. V. Frolov, P. Fulda, M. Fyffe, B. Gateley, J. A. Giaime, K. D. Giardina, J. Glanzer, E. Goetz, R. Goetz, A. W. Goodwin-Jones, S. Gras, C. Gray, D. Griffith, H. Grote, T. Guidry, E. D. Hall, J. Hanks, J. Hanson, M. C. Heintze, A. F. Helmling-Cornell, N. A. Holland, D. Hoyland, H. Y. Huang, Y. Inoue, A. L. James, A. Jennings, S. Karat, S. Karki, M. Kasprzack, K. Kawabe, P. J. King, J. S. Kissel, K. Komori, A. Kontos, R. Kumar, K. Kuns, M. Landry, B. Lantz, M. Laxen, K. Lee, M. Lesovsky, F. Llamas, M. Lormand, H. A. Loughlin, R. Macas, M. MacInnis, C. N. Makarem, B. Mannix, G. L. Mansell, R. M. Martin, K. Mason, F. Matichard, N. Mavalvala, N. Maxwell, G. McCarrol, R. McCarthy, D. E. McClelland, S. McCormick, T. McRae, F. Mera, E. L. Merilh, F. Meylahn, R. Mittleman, D. Moraru, G. Moreno, T. J. N. Nelson, A. Neunzert, J. Notte, J. Oberling, T. O'Hanlon, C. Osthelder, D. J. Ottaway, H. Overmier, W. Parker, A. Pele, H. Pham, M. Pirello, V. Quetschke, K. E. Ramirez, J. Reyes, J. W. Richardson, M. Robinson, J. G. Rollins, C. L. Romel, J. H. Romie, M. P. Ross, K. Ryan, T. Sadecki, A. Sanchez, E. J. Sanchez, L. E.

Sanchez, R. L. Savage, D. Schaetzl, M. G. Schiworski, R. Schnabel, R. M. S. Schofield, E. Schwartz, D. Sellers, T. Shaffer, R. W. Short, D. Sigg, B. J. J. Slagmolen, C. Soike, S. Soni, V. Srivastava, L. Sun, D. B. Tanner, M. Thomas, P. Thomas, K. A. Thorne, C. I. Torrie, G. Traylor, A. S. Ubhi, G. Vajente, J. Vanosky, A. Vecchio, P. J. Veitch, A. M. Vibhute, E. R. G. Von Reis, J. Warner, B. Weaver, R. Weiss, C. Whittle, B. Willke, C. C. Wipf, H. Yamamoto, L. Zhang, M. E. Zucker, LIGO O4 Detector Collaboration, Broadband quantum enhancement of the LIGO detectors with frequency-dependent squeezing. *Phys. Rev. X* **13**, 041021 (2023).

47. W. Jia, V. Xu, K. Kuns, M. Nakano, L. Barsotti, M. Evans, N. Mavalvala, members of the LIGO Scientific Collaboration, Squeezing the quantum noise of a gravitational-wave detector below the standard quantum limit. *Science* **385**, 1318–1321 (2024).

48. F. Wolfgramm, A. Cerè, F. A. Beduini, A. Predojević, M. Koschorreck, M. W. Mitchell, Squeezed-light optical magnetometry. *Phys. Rev. Lett.* **105**, 053601 (2010).

49. C. Troullinou, R. Jiménez-Martínez, J. Kong, V. G. Lucivero, M. W. Mitchell, Squeezed-light enhancement and backaction evasion in a high sensitivity optically pumped magnetometer. *Phys. Rev. Lett.* **127**, 193601 (2021).

50. S. Wu, G. Bao, J. Guo, J. Chen, W. Du, M. Shi, P. Yang, L. Chen, W. Zhang, Quantum magnetic gradiometer with entangled twin light beams. *Sci. Adv.* **9**, eadg1760 (2023).

51. C. A. Casacio, L. S. Madsen, A. Terrasson, M. Waleed, K. Barnscheidt, B. Hage, M. A. Taylor, W. P. Bowen, Quantum-enhanced nonlinear microscopy. *Nature* **594**, 201–206 (2021).

52. B. Yurke, S. L. McCall, J. R. Klauder, SU(2) and SU(1,1) interferometers. *Phys. Rev. A* **33**, 4033–4054 (1986).

53. F. Hudelist, J. Kong, C. Liu, J. Jing, Z. Y. Ou, W. Zhang, Quantum metrology with parametric amplifier-based photon correlation interferometers. *Nat. Commun.* **5**, 3049 (2014).

54. B. E. Anderson, P. Gupta, B. L. Schmittberger, T. Horrom, C. Hermann-Avigliano, K. M. Jones, P. D. Lett, Phase sensing beyond the standard quantum limit with a variation on the SU(1,1) interferometer. *Optica* **4**, 752 (2017).

55. M. Manceau, G. Leuchs, F. Khalili, M. Chekhova, Detection loss tolerant supersensitive phase measurement with an SU(1,1) interferometer. *Phys. Rev. Lett.* **119**, 223604 (2017).

56. W. Du, J. Kong, G. Bao, P. Yang, J. Jia, S. Ming, C.-H. Yuan, J. F. Chen, Z. Y. Ou, M. W. Mitchell, W. Zhang, SU(2)-in-SU(1,1) nested interferometer for high sensitivity, loss-tolerant quantum metrology. *Phys. Rev. Lett.* **128**, 033601 (2022).

57. W. Du, S. Wu, D. Zhang, J. Chen, Y. Yang, P. Yang, J. Guo, G. Bao, W. Zhang, Quantum twin Interferometers. arXiv:2501.04244 [physics.atom-ph] (2025).

58. R. C. Pooser, N. Savino, E. Batson, J. L. Beckey, J. Garcia, B. J. Lawrie, Truncated nonlinear interferometry for quantum-enhanced atomic force microscopy. *Phys. Rev. Lett.* **124**, 230504 (2020).

59. V. Boyer, A. M. Marino, R. C. Pooser, P. D. Lett, Entangled images from four-wave mixing. *Science* **321**, 544–547 (2008).

60. E. Brambilla, L. Caspani, O. Jedrkiewicz, L. A. Lugiato, A. Gatti, High-sensitivity imaging with multi-mode twin beams. *Phys. Rev. A* **77**, 053807 (2008).

61. G. Brida, M. Genovese, I. Ruo Berchera, Experimental realization of sub-shot-noise quantum imaging. *Nat. Photonics* **4**, 227–230 (2010).

62. S.-H. Tan, B. I. Erkmen, V. Giovannetti, S. Guha, S. Lloyd, L. Maccone, S. Pirandola, J. H. Shapiro, Quantum illumination with gaussian states. *Phys. Rev. Lett.* **101**, 253601 (2008).

63. E. D. Lopaeva, I. Ruo Berchera, I. P. Degiovanni, S. Olivares, G. Brida, M. Genovese, Experimental realization of quantum illumination. *Phys. Rev. Lett.* **110**, 153603 (2013).

64. Z. Zhang, M. Tengner, T. Zhong, F. N. C. Wong, J. H. Shapiro, Entanglement's benefit survives an entanglement-breaking channel. *Phys. Rev. Lett.* **111**, 010501 (2013).

65. Z. Zhang, S. Mouradian, F. N. C. Wong, J. H. Shapiro, Entanglement-enhanced sensing in a lossy and noisy environment. *Phys. Rev. Lett.* **114**, 110506 (2015).

66. J. Liu, Q. Yang, S. Chen, Z. Xiao, S. Wen, H. Luo, Intrinsic optical spatial differentiation enabled quantum dark-field microscopy. *Phys. Rev. Lett.* **128**, 193601 (2022).

67. T. Li, V. Cheburkanov, V. V. Yakovlev, G. S. Agarwal, M. O. Scully, Harnessing quantum light for microscopic biomechanical imaging of cells and tissues. *Proc. Natl. Acad. Sci. U.S.A.* **121**, e2413938121 (2024).

68. Y. Zhang, Z. He, X. Tong, D. C. Garrett, R. Cao, L. V. Wang, Quantum imaging of biological organisms through spatial and polarization entanglement. *Sci. Adv.* **10**, eadk1495 (2024).

69. Y. Chen, Y. Qiu, M. D. Lew, Resolving the orientations of and angular separation between a pair of dipole emitters. *Phys. Rev. Lett.* **134**, 093805 (2025).

70. W. P. Bowen, N. Treps, R. Schnabel, P. K. Lam, Experimental demonstration of continuous variable polarization entanglement. *Phys. Rev. Lett.* **89**, 253601 (2002).

71. W. P. Bowen, R. Schnabel, H.-A. Bachor, P. K. Lam, Polarization squeezing of continuous variable stokes parameters. *Phys. Rev. Lett.* **88**, 093601 (2002).

72. L. D. Barron, *Molecular Light Scattering and Optical Activity-Cambridge University Press* (Cambridge Univ. Press, 2024).

73. K. C. Toussaint, G. Di Giuseppe, K. J. Bycenski, A. V. Sergienko, B. E. A. Saleh, M. C. Teich, Quantum ellipsometry using correlated-photon beams. *Phys. Rev. A* **70**, 023801 (2004).

74. A. Belsley, J. C. F. Matthews, Estimating the concentration of chiral media with bright squeezed light. *Appl. Phys. Lett.* **121**, 184001 (2022).

75. Y. Chen, S. Ecker, S. Wengerowsky, L. Bulla, S. K. Joshi, F. Steinlechner, R. Ursin, Polarization entanglement by time-reversed Hong-Ou-Mandel interference. *Phys. Rev. Lett.* **121**, 200502 (2018).

76. R. Camphausen, Á. Cuevas, L. Duempelmann, R. A. Terborg, E. Wajs, S. Tisa, A. Ruggeri, I. Cusini, F. Steinlechner, V. Pruneri, A quantum-enhanced wide-field phase imager. *Sci. Adv.* **7**, eabj2155 (2021).

77. J. Ceramella, D. Iacopetta, A. Franchini, M. De Luca, C. Saturnino, I. Andreu, M. S. Sinicropi, A. Catalano, A look at the importance of chirality in drug activity: Some significative examples. *Appl. Sci.* **12**, 10909 (2022).

78. T. Akiyama, I. Ojima, *Catalytic Asymmetric Synthesis* (John Wiley & Sons, 2022).

79. T. Buhse, J.-M. Cruz, M. E. Noble-Terán, D. Hochberg, J. M. Ribó, J. Crusats, J.-C. Micheau, Spontaneous deracemizations. *Chem. Rev.* **121**, 2147–2229 (2021).

80. W. L. Noorduin, A. A. C. Bode, M. Van Der Meijden, H. Meekes, A. F. Van Etteger, W. J. P. Van Enckevort, P. C. M. Christianen, B. Kaptein, R. M. Kellogg, T. Rasing, E. Vlieg, Complete chiral symmetry breaking of an amino acid derivative directed by circularly polarized light. *Nat. Chem.* **1**, 729–732 (2009).

81. M. Deng, J. Yu, D. G. Blackmond, Symmetry breaking and chiral amplification in prebiotic ligation reactions. *Nature* **626**, 1019–1024 (2024).

82. K. J. Resch, K. L. Pregnell, R. Prevedel, A. Gilchrist, G. J. Pryde, J. L. O'Brien, A. G. White, Time-reversal and super-resolving phase measurements. *Phys. Rev. Lett.* **98**, 223601 (2007).

83. J. Qin, Y.-H. Deng, H.-S. Zhong, L.-C. Peng, H. Su, Y.-H. Luo, J.-M. Xu, D. Wu, S.-Q. Gong, H.-L. Liu, H. Wang, M.-C. Chen, L. Li, N.-L. Liu, C.-Y. Lu, J.-W. Pan, Unconditional and robust quantum metrological advantage beyond N00N states. *Phys. Rev. Lett.* **130**, 070801 (2023).
